# Paroxysmal Nocturnal Hemoglobinuria: Biology and Treatment

**DOI:** 10.3390/medicina59091612

**Published:** 2023-09-06

**Authors:** Carlos Bravo-Perez, Luca Guarnera, Nakisha D. Williams, Valeria Visconte

**Affiliations:** 1 Department of Translational Hematology & Oncology Research, Taussig Cancer Institute, Cleveland Clinic, Cleveland, OH 44114, USA; bravoc2@ccf.org (C.B.-P.); guarnel@ccf.org (L.G.); willian28@ccf.org (N.D.W.); 2 Department of Hematology and Medical Oncology, Hospital Universitario Morales Meseguer, IMIB-Pascual Parrilla, CIBERER—Instituto de Salud Carlos III, University of Murcia, 30005 Murcia, Spain; 3 Hematology, Department of Biomedicine and Prevention, University of Rome Tor Vergata, 00133 Rome, Italy

**Keywords:** paroxysmal nocturnal hemoglobinuria, biology, treatment

## Abstract

Paroxysmal nocturnal hemoglobinuria (PNH) is a nonmalignant clonal hematopoietic disorder characterized by the lack of glycosylphosphatidylinositol-anchored proteins (GPI-APs) as a consequence of somatic mutations in the phosphatidylinositol glycan anchor biosynthesis class A (*PIGA*) gene. Clinical manifestations of PNH are intravascular hemolysis, thrombophilia, and bone marrow failure. Treatment of PNH mainly relies on the use of complement-targeted therapy (C5 inhibitors), with the newest agents being explored against other factors involved in the complement cascade to alleviate unresolved intravascular hemolysis and extravascular hemolysis. This review summarizes the biology and current treatment strategies for PNH with the aim of reaching a general audience with an interest in hematologic disorders.

## 1. Introduction

Paroxysmal nocturnal hemoglobinuria (PNH) is a rare hematologic disorder. The incidence is estimated to be about one per million, and the prevalence is about eight per million in the United States [1]. It is a disease of adults. In fact, only 5–10% of PNH patients are pediatric [2,3,4]. Somatic mutations in the X-linked phosphatidylinositol glycan anchor biosynthesis class A (*PIGA*) gene cause complete or partial deficiency of glycosylphosphatidil-anchored proteins (GPI-APs), a hallmark biological manifestation of PNH. The loss of the GPI-AP CD55 (decay-accelerating factor for complement) and CD59 (membrane attack complex [MAC] inhibition factor) makes PNH erythrocytes sensitive to intravascular hemolysis and thrombosis since their role is to protect red blood cells from complement-mediated lysis. The expansion of abundant clones characterized by the lack of GPI-APs is associated with hemolytic signs of PNH. PNH clones can also be found in patients with aplastic anemia (AA), a bone marrow failure disorder triggered by the attack of autoreactive cytotoxic T lymphocytes on the hematopoietic stem cell (HSC) compartment [5]. The co-existence of PNH clones in AA has been attributed to the presence of GPI-APs escaping immune attack. Patients with AA and small PNH clones are more likely to respond to immunosuppressive treatment [6]. *PIGA* mutations have been found in granulocytes of healthy individuals with minor PNH clones occurring between 0.001 and 0.005% without leading to any symptoms [7,8]. An open question remains on why PNH clones expand.

## 2. Biology and Pathogenesis of PNH

### 2.1. Clonal Expansion in PNH and Mechanisms of Immune Escape

The pathogenesis of PNH is at the center of a long-lasting debate. The common association of PNH with AA (about 10% of AA patients develop PNH during their follow-up) has led to the most accepted theory, which attributes the clonal expansion to a survival advantage of PNH HSCs during autoimmune attacks, with subsequent growth of the PNH clone once the inflammatory reaction is controlled with immunosuppression [9].

The precise nature of the postulated survival advantage is a key, still not fully clarified question, given the theory suggesting that such advantage is simply due to neutral drift [10].

The peculiar features of the PNH clone (nonmalignant, able to coexist with normal hematopoietic cells without a selective fitness advantage, detectable at a low fraction in healthy subjects) have prompted researchers to attribute this advantage to the inherent resistance of PNH cells to apoptosis. In the late 1990s, Brodsky and colleagues observed that granulocytes and CD34(+)CD59(−) cells from PNH patients had longer survival compared to their normal counterparts when placed in a serum-free medium. Furthermore, PNH cells were also relatively resistant to apoptosis induced by ionizing irradiation [11]. In the same line of research, Savage and colleagues documented a higher resistance rate of apoptosis in response to TNFα or gamma radiation in GPI-AP(−) cells when compared to the isogenic GPI-AP(+) counterparts [12], while Kunyaboon et al. detected a higher survival rate of CD59(−) granulocytes after 0 or 4 h in liquid growth culture system compared to that of CD59(+) [13]. Furthermore, a study identified four anti-apoptosis genes (human A1, hHR23B, Mcl-1, and RhoA) overexpressed in peripheral blood granulocytes and mononuclear cells of patients with PNH compared to healthy controls [14]. Subsequently, it confirmed the relative resistance to apoptosis of granulocytes from patients with PNH as compared with normal granulocytes, but this resistance did not correlate with the level of expression of GPI-APs [15]. Analysis of CD34(+) cells from PNH patients demonstrated a higher apoptosis rate of CD55(+)CD59(+) cells when compared to GPI-AP-deficient ones and to normal CD34(+) cells, suggesting frailty of progenitor cells in PNH patients more than an advantage of PNH cells [16,17]. Finally, Yamamoto et al. and Kulkarni et al. published diametrically opposed results, showing no difference in apoptosis sensitivity between PNH granulocytes and normal controls [18,19].

Together, these studies suggest that the mechanism underlying the survival advantage of PNH is beyond the controversial inherent resistance of PNH cells to apoptosis stimuli and may lie in the selection of GPI-AP(−) cells in a hostile marrow environment of PNH patients.

The presence of additional molecular lesions has been largely investigated as an intrinsic factor conferring a survival advantage. A small proportion of PNH cases have been found carrying microdeletions of the *PIGA* locus or concurrent lesions. In one study, two PNH patients were characterized by an acquired rearrangement of chromosome 12 (der(12) had a break within the 3′ untranslated region of *HMGA2* producing an ectopic expression of HMGA2) [20], and in another study, three PNH patients were found harboring a microdeletion of Xp22.2, encompassing the *PIGA* locus [21]. Other scattered somatic mutations have also been identified in non-PNH [22] and PNH cells or co-occurrence of mutations in PNH clones [17,23,24].

Whole exome and deep next-generation sequencing were used to investigate the possibility that other molecular lesions may increase PNH cell fitness. In-depth analysis showed that HSCs of PNH patients have other hits apart from the one in the *PIGA* gene (*JAK2*, *SUZ12*, *TET2*, *U2AF1*) [25].

Similarly, polymorphisms in ABO (genotypes with minor alleles of rs495828 or rs2519093) have been associated with an increased risk of thrombosis in PNH. Correlation between the risk of thrombosis, larger PNH clone, and minor alleles of rs495828 or rs2519093 of ABO was found. In addition, higher levels of vWF and factor VIII were found in patients carrying the minor allele compared with those carrying the major allele [26]. Alterations in non-GPI-APs have also been detected in PNH, possibly explaining the key involvement of main regulators of the complement alternative pathway. This is the case of rare germline variants in complement factor H (CFH). Patients carrying germline CFH variants were more likely to be transfusion-dependent at 6 months after eculizumab initiation. Out of nine patients with CFH variants, two developed thrombosis [27].

**Figure 1:**
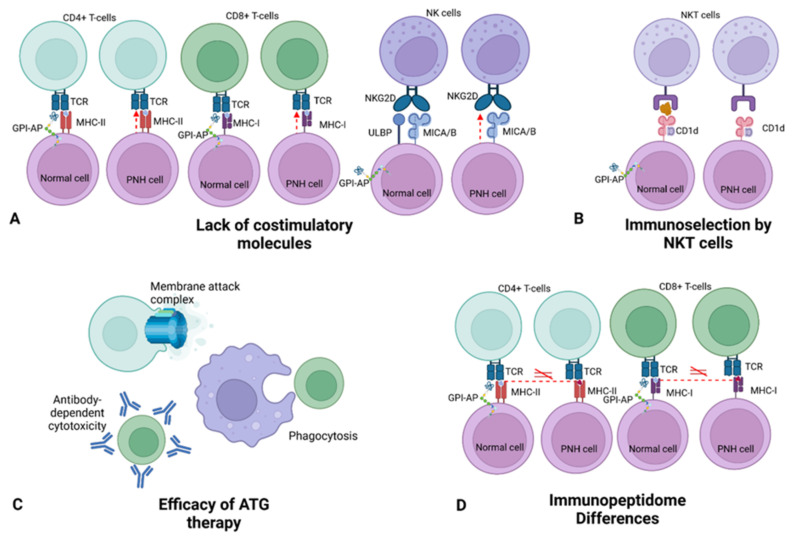
Evidence and theories on PNH immunoselection. (**A**) Lack of costimulation in cell-to-cell interactions in T and NK cells. In the latter case, ULBP proteins, GPI-APs lacking in PNH, and MICA/B were identified as ligands of NKG2D NK cell receptor. (**B**) NKT cells are able to recognize lipid antigens from GPI-AP molecules, inherent lacking in PNH, presented on the surface of the cells through CD1d receptor. (**C**) Efficacy of anti-human thymocyte globulin (ATG) therapy. Main mechanisms of action of ATG include complement-dependent cytotoxicity, with the formation of the membrane attack complex (MAC), opsonization process, subsequent phagocytosis, and antibody-dependent cytotoxicity. (**D**) The discovery of several HLA combinations differently expressed between PNH patients and controls and the detection of mutations in HLA machinery in *PIGA*-mutated cells led to the hypothesis of the role of the immunopeptidome in the pathogenesis of PNH. Figure was generated with BioRender.com, accessed on 8 July 2023.

Although extensive research efforts have been invested in explaining the survival advantage of PNH cells, currently, it has been hypothesized that the solution to the survival advantage of PNH HSCs enigma can lie in the complex immunologic interactions and in the microenvironment changes in the context of autoimmunity response (Figure 1).

### 2.2. Immunoselection by Effector Cells

Besides the contact between the receptor of the effector cells (e.g., T and NK cells) and the target antigen, bonded to protein-expressing machinery on the surface of presenting cells, a key point of the process of activation and functioning of innate and acquired immunity is the link with costimulatory molecules [28] at the center of several studies and theories on the pathogenesis of PNH.

Some of the first clear evidence of the potential role of these molecules was provided by the experiments of Murakami et al., who, in the early 2000s, comparing isogenic GPI-AP(+) and GPI-AP(−) cell lines, showed not only that the second ones were unable to present GPI-AP antigens to CD4(+) T-cells but also that, when presenting non-GPI-AP antigens, the stimulation was significantly weaker than the one by the GPI-AP(+) counterpart [29]. These findings suggest a role of costimulatory molecules in the cell-to-cell interaction differences between *PIGA*-mutated and *PIGA*–wild type (WT) cells. The nature of the costimulation was identified by Deckert et al. in two signaling pathways: the first one involving ZAP-70 activation and leading to IL-2 secretion, and a second pathway was observed in the absence of ZAP-70 activation leading to CD25 expression [30]. Several studies, on the other hand, highlighted a higher expression of Fas, part of the Fas/FasL pathway able to induce apoptosis in target cells, in CD59(+) cells than in PNH cells [16,31,32], supporting the thesis of a Fas-dependent T-cell attack through GPI-APs [33].

In a similar fashion, Nagakura et al. documented resistance of *PIGA*-mutated cells to perforin-mediated NK cytotoxicity when compared to control cells. Of note, in the presence of soluble perforin, PNH cells were lysed similarly to controls [34]. This finding suggested a similar role of costimulatory molecules in NK cells. In this case, the nature of PNH cells advantage was found some years later in the lack of a specific GPI-AP family on the surface of PNH cells: ULBP, stress-inducible GPI-linked membrane proteins able to bind an NKG2D receptor, along with MICA and MICB, on NK cells. Hanaoka et al., in fact, not only documented ULBP expressed only in *PIGA*-WT cells and not in the mutated ones but also showed that, after blocking NKG2D or ULBP with antibodies, both controls cells and PNH cells presented resistance to NK cell attacks [35] (Figure 1A).

An alternative thesis, proposed by Rotoli et al., conceived an autoimmune attack of effector cells directly stimulated by GPI-AP molecules [36]. Two studies supported this theory by identifying a specialized subtype of lymphoid cells, the natural killer T-cells (NKT cells), able to recognize a specific lipid antigen-presenting receptor, CD1d [37,38] (Figure 1B). However, the lack of a correlation between the number of CD1d-restricted lymphocytes and the size of the PNH clone and the evidence that not all AA patients presented this peculiar kind of effector cells challenged this thesis [37,39].

Considering the extensive evidence that shows that autoimmune attacks in AA are mainly due to CD8(+) T-cell reaction [40,41] and the studies by Gargiulo et al., who analyzed the receptors’ structure and found similar sequences in CD8(+) T-cells from PNH patients but not from normal controls, supporting their role in the autoimmune attack [42], it can be assumed that T-cell immune escape mechanisms are the major characters in PNH selection. Indeed, the TCR-β repertoire is skewed mainly among CD8+ T-cells. This study proved that using size analysis of TCR-β gene CDR3 coupled with shotgun cloning, nearly identical CDR3 sequences were present in CD8+ CD57+ T-cells of more than half of patients with full-blown hemolytic PNH. Such type of lymphocytes was similar in size to the ones of healthy controls but showed an oligoclonal configuration of abnormal/ expanded T-cell clones. These results pointed out an immune process possibly driven by the same antigen or a group of related antigens.

Nevertheless, one can hypothesize that the NK cell resistance mechanism can cooperate in the process and have higher importance in peculiar cases, such as PNH evolving without a previous phase of AA.

Finally, as a posteriori proof of the involvement of T-cells in the pathogenesis of PNH, ATG treatment showed efficacy in both AA/PNH and PNH patients [43,44,45] (Figure 1C).

### 2.3. Role of HLA

As mentioned above, the activation of T-cells goes through contact with major histocompatibility complex (MHC; in humans, called the human leukocyte antigen [HLA]), molecules capable of binding short peptides derived from intracellular proteins cleaved by the proteasome and peptidases and display them on cell surface. MHC class I (HLA-A, B, C) is expressed in most nucleated cells, whereas MHC class II (HLA-DR, DQ, DP) is expressed mostly in immune system cells [46].

There are over 25,000 HLA class I and 10,000 HLA class II known alleles, which encode for about 14,000 HLA class I and 9000 HLA class II protein allomorphs. Since each HLA haplotype presents thousands of peptides, the entire repertoire of the presented peptides (immunopeptidome) is extremely large [46].

In this broad landscape of possible combinations, several efforts have been made to correlate AA and the emergence of PNH to a specific HLA class or haplotype expression in a fashion similar to the one adopted to investigate the well-known HLA association with other autoimmune diseases [47,48,49,50,51,52,53,54] (Table 1).

Despite the inter-study discrepancies, most likely due to the specific geographic areas [55], the identification of several HLA combinations differently expressed between PNH patients and controls underpins the central role of HLA in the pathogenesis of the disease.

Pagliuca and colleagues also investigated peptide presentation, which showed a correlation between *PIGA* and HLA somatic gene mutations. Furthermore, a comparison between AA patients’ bone marrow and hematopoietic stem cells revealed an upregulation of the gene involved in HLA antigen machinery, processing, and regulation (among others, proteasome subunits and genes involved in endoplasmic reticulum-mediated exocytosis) [56].

Taken together, this evidence suggests that PNH selection may be due to differences in immunopeptidome (Figure 1D): the antigenic presentation changes may be due to a different HLA class and haplotypes expression by *PIGA* mutant cells and the acquisition of HLA mutations. However, other scenarios are conceivable.

For example, one can hypothesize that the inherent nature of *PIGA* cells and the subsequent lack of expression of GPI-APs can lead to an intracellular accumulation of proteins. The cleavage by the proteasome, upregulated as found by the above-mentioned recent studies, and exposure of these unusual antigens can both be unrecognized by autoreactive T-cells and dilute the trigger antigen, thus promoting immune escape.

## 3. Treatment

Historically, the treatment of PNH was exclusively supportive, and the median survival of patients was 15–20 years, thrombosis being the major cause of death [57,58]. Supportive measures included corticosteroids during hemolytic attacks, androgen therapy, and red blood cell transfusions for the treatment of anemia, as well as anticoagulant therapy to treat/prevent thrombotic events. However, the use of these palliative measures was not evidence-based, their effectiveness was limited, and they were not devoid of adverse events. Allogeneic HSC transplant was and continues to be, indeed, the only curative treatment of PNH, but it is reserved for young patients with concurrent bone marrow failure because of transplant-related morbidity and mortality [59].

With the advent of complement inhibitors in clinical practice, the survival of patients with PNH has become close to that of sex- and age-matched controls [60,61]. Complement inhibitors are indicated for the treatment of classic hemolytic PNH. Indications to start treatment in this setting include thrombosis, debilitating fatigue, transfusion dependence, pain paroxysms, and organ complications. Asymptomatic patients should be carefully watched. The use of complement inhibition should be accompanied by vaccination against *Neisseria* and penicillin, given the risk of infection.

Eculizumab, the first developed complement factor inhibitor, inhibits C5, blocking the terminal complement cascade. Given the benefits of the complement inhibition strategy, changing the natural history of this disease, several novel drugs against C5 (terminal inhibitors) or against factors upstream C5 (proximal inhibitors) have been developed with the aim of further improving the treatment of PNH. Eculizumab, ravulizumab, and pegcetacoplan are the three complement inhibitors currently approved by the US Food and Drug Administration (FDA) and the European Medicines Agency (EMA) for the treatment of PNH. Approved and upcoming complement inhibitors are discussed below.

### 3.1. Terminal Complement Factor Inhibitors

Complement factor C5 was initially identified as the optimal target for PNH therapeutics. Firstly, because C5 cleavage is the common endpoint of the classical, alternative, and lectin pathways, C5 inhibition may render effective blocking of the complement cascade, regardless of the triggers. Secondly, there were initial concerns about the potential deleterious effects of inhibition upstream C5 on the immune-regulatory roles of complement [60].

#### 3.1.1. Eculizumab and Ravulizumab

Eculizumab, FDA- and EMA-approved in 2007, was the first complement factor inhibitor developed for the treatment of PNH. It is a humanized anti-C5 monoclonal antibody (mAb) that prevents C5 cleavage into C5a and C5b. After a first pilot study, demonstrating that it inhibited intravascular hemolysis in PNH [62], TRIUMPH (NCT00122330) [63], and SHEPERD (NCT0013000) [64], two large phase III, placebo-controlled, randomized controlled trials (RCTs) showed that eculizumab was safe and effective, leading to the stabilization of hemoglobin levels and reduction in transfusion requirements. Studies on the long-term use of eculizumab and real-world data studies have also shown a significant drop in the rate of thrombotic events, thus explaining the change in the life expectancy of patients with PNH. The safety and efficacy of eculizumab have also been demonstrated in special situations, such as pregnancy and pediatric patients [65,66].

Despite the substantial efficacy of eculizumab, which revolutionized PNH, several areas of improvement have been rapidly identified. The short half-life of eculizumab requires a frequent dosing schedule (IV infusion/2 weeks), and it is associated with an increased risk of breakthrough hemolysis due to insufficient complement inhibition 24 to 48 h before the next infusion. Consequently, novel long-acting C5 inhibitors have been developed to address many of these pharmacokinetic (PK) limitations [67].

Ravulizumab, FDA- and EMA-approved in 2018 and 2019 for eculizumab-treated or naïve patients, is a humanized anti-C5 mAb engineered from eculizumab to achieve a longer half-life. By the introduction of only four aminoacidic substitutions, the kinetics of binding of ravulizumab were selectively changed, favoring its dissociation from C5 in the early endosome after the uptake of C5-Ab complexes, as well as their recycling back to the vascular system through increased binding to the neonatal Fc receptor (FcRn). Based on the positive results of the phase I/II studies, two large phase III trials were conducted, one in patients already treated with eculizumab (NCT03056040) [68] and the other in treatment-naïve PNH patients (NCT02946463) [69]. Both studies concluded that ravulizumab was non-inferior as compared to eculizumab in terms of hemolysis control and transfusion avoidance. Adverse events were similar between both treatment arms.

Administered by IV infusion every 8 weeks during the maintenance phase, ravulizumab is replacing eculizumab in an increasing number of countries. In addition, an SC formulation of ravulizumab for once-weekly self-administration with an on-body infusion device has been developed and recently shown to be non-inferior to IV ravulizumab in terms of PK and efficacy [70].

#### 3.1.2. Other C5 Inhibitors

Crovalimab is a long-acting anti-C5 mAb targeting a different epitope of C5 than eculizumab/ravulizumab (thus, not susceptible to the rare problem of intrinsic resistance described for the former ones due to C5 polymorphisms) [71], which is in the advanced stage of development. Administered SC every 4 weeks, with the option to self-administer, crovalimab is currently being studied in the phase III COMMODORE trials [72,73,74]. Preliminary results show that, in both eculizumab-exposed and naïve patients, crovalimab is non-inferior to eculizumab for the control of hemolysis and transfusion avoidance. There are no relevant safety concerns. Transient, non-severe type III hypersensitivity events unique to patients switching between crovalimab and eculizumab were recently reported in the COMMODORE 1 trial, but the overall favorable benefit/risk profile for crovalimab in both treated and untreated patients suggests that it might join ravulizumab in the near future as a C5 inhibitor of choice for PNH [72,73].

Multiple other C5 inhibitors have been developed for the treatment of PNH, including classes of drugs other than mAbs. However, not all of them have proceeded to phase III trials, as has been the case for tesidolumab, zilucoplan, and nomacopan. Cemdiseran is a novel small-interfering RNA (siRNA) directed against C5, conjugated to N-acetylgalactosamine. Administered SC, it acts as a liver-targeted suppressor of C5 synthesis. Although it has been proven to achieve an effective and unprecedented sustained reduction of C5 (up to 13 months), induction of response is mechanistically retarded, and severe breakthrough hemolysis has been observed in monotherapy. Thus, a combinatory approach based on cemdiseran plus the mAb pozelimab has been proposed for complete and durable inhibition, which is being tested in an ongoing phase III trial.

### 3.2. Limitations of C5 Inhibition: Extravascular Hemolysis

C5 inhibitors after eculizumab offer the benefit of a more favorable posology likely to reduce treatment burden and improve patients’ quality of life. In addition, they prevent PK intravascular breakthrough hemolysis, which was observed with eculizumab in a relevant proportion of cases. Nevertheless, there are still several points of improvement in the treatment of hemolytic PNH. About 25% of cases still need blood transfusions. Causes of residual anemia may include nutrient deficiencies or inefficient erythropoiesis due to concomitant bone marrow failure. However, the most relevant cause of residual anemia under terminal inhibitors may be “iatrogenic”, secondary to the inhibition of C5 by itself. This is the case of C3-mediated extravascular hemolysis seen in patients treated with C5 inhibitors. Anti-C5 drugs effectively prevent MAC assembly and the intravascular lysis of PNH erythrocytes. However, upon C5 inhibition, there is still uncontrolled C3 convertase activity due to constitutive activation of the alternative pathway and the lack of CD55. Activation of C3 in the surface of red blood cells leads to the deposition of C3b and then C3d, which eventually results in the opsonization and premature destruction of PNH erythrocytes. The result is residual, extravascular hemolytic anemia that can turn positive for the C3 direct antiglobulin test. This collateral effect, highly variable among patients, may occur in virtually all cases and be universal for anti-C5 drugs, becoming a clinically relevant problem unlikely to be overcome by novel terminal inhibitors [67,75].

### 3.3. The Emergence of Proximal Complement Inhibitors

With this background, the interest in drug discovery in PNH was re-directed upstream C5. Complement factor C3 became the next target. Among the different pharmacological approaches developed for the inhibition of C3, pegcetacoplan, a compstatin-based C3 inhibitor is the third treatment for PNH that has been approved by regulatory agencies. In addition, other inhibitors targeting the alternative pathway are showing promising results and rapidly moving forward with the potential of changing PNH clinical practice.

#### 3.3.1. Pegcetacoplan

Pegcetacoplan, approved in 2021 by the FDA for C5 inhibitor–exposed or naïve patients and by the EMA for patients refractory to C5 inhibitors, is a pegylated compstatin derivative that binds to C3 in the native and C3b forms, both preventing its cleavage into C3a and C3b as well as generating surface C3 effectors. Administered SC twice weekly after positive results from phase I/II trials [76], pegcetacoplan was tested in two phase III RCTs, assessing its efficacy and safety vs. eculizumab in eculizumab-treated patients (PEGASUS trial, NCT03500549) [77] and vs. standard of care not including complement inhibitors in treatment-naïve patients (NCT04085601) [78]. Both studies revealed that pegcetacoplan was superior to the control arms, including superiority to eculizumab in the PEGASUS trial by improving hematological and other clinical responses as early as in week 2, which persisted at the end of the 32-week open-label period. The safety profile was acceptable in the pegcetacoplan arm. In the 48-week follow-up analysis, hemolysis was the most frequent adverse event (19%) after injection site reactions. Three of forty-one patients (7%) had severe episodes of breakthrough hemolysis leading to treatment discontinuation through to week 16 [79], and during the entire study, there were 13 out of 80 (16%) interruptions of treatment because of this and other adverse events. Breakthrough hemolysis in this context has been attributed to an unprecedented increase in the percentage of PNH red blood cells. Although with an increased survival, this population is still highly susceptible to complement. Thus, unlike residual extravascular hemolysis under terminal inhibitors, massive breakthrough intravascular hemolysis under pegcetacoplan (and potentially other proximal inhibitors) may arise from transient episodes of incomplete complement inhibition because of PK reasons or overwhelming activations because of irruptive triggers. Despite these considerations, pegcetacoplan in monotherapy was able to improve hematologic response in poor responders to eculizumab by preventing C3-mediated extravascular hemolysis while controlling MAC-mediated intravascular hemolysis at an acceptable level, supporting its approval by the regulatory agencies [67].

#### 3.3.2. Other Upcoming Proximal Inhibitors

Encouraged by the success of pegcetacoplan, numerous proximal inhibitors, not only against C3 but also targeting elements of the complement alternative pathway upstream of C3, have been generated, some of which are in the advanced stage of development for the treatment of PNH. This is the case of small molecules against factors D (FD) and B (FB) (Table 2).

Danicopan, vemircopan, and BCX9930 are oral inhibitors of FD. Iptacopan is an oral inhibitor of FB. Except for vemircopan, early clinical trials on these drugs have been (or are being) conducted in combination with C5 inhibitors for patients with PNH and extravascular hemolysis or inadequate response to this therapy. In addition, vemircopan, BCX9930, and iptacopan have been (or are being) evaluated in monotherapy for previously treated and/or untreated patients.

The combination of terminal and proximal inhibitors has the rationale to provide a more robust control and thus prevent both intravascular and extravascular hemolysis. Severe episodes of breakthrough hemolysis due to incomplete proximal inhibition may also be less likely with this strategy. Danicopan has proceeded through this approach. The ALPHA trial is a phase III, double-blind RCT designed as a superiority study to assess the efficacy and safety of add-on danicopan vs. placebo in patients receiving eculizumab or ravulizumab. While the study has not been completed, the first results recently communicated suggest that the combination with danicopan significantly improves hematological responses by addressing extravascular hemolysis while maintaining control of intravascular hemolysis with a favorable benefit/risk profile [80]. If these findings are confirmed, benefit/cost analyses of combinatory vs. other single-agent-based strategies may additionally be considered. Conversely, KP104, a novel and first-in-class bi-functional complement inhibitor, has been developed to simultaneously achieve inhibition of the terminal and proximal pathways with a single agent. KP104 is an engineered anti-C5 mAb with an FH-based peptide in the Fc region, targeting the C5 as well as the C3 convertase, respectively. A proof-of-concept trial with this innovative, bi-specific agent in naïve PNH patients is ongoing (NCT05476887).

As monotherapy, FD inhibitors vemircopan and BCX9930 have been (or are) in phase II studies, and the FB inhibitor iptacopan (supported by favorable results in combination with C5 inhibitors) [81] has been tested in phase III trials. Vemircopan is currently in a phase II study (NCT04170023) with three arms, including patients treated with eculizumab and inadequate response, subjects treated with danicopan in monotherapy from ACH471-103 study (NCT03181633), and naïve patients. The study is ongoing, but an interim analysis from previously untreated patients (*n* = 11) preliminarily supported safety and efficacy [82]. On the contrary, the development of BCX9930 was recently discontinued by the company in favor of BCX10013 [83], another FD inhibitor with best-in-class potential, currently in the early research stage.

Iptacopan, the first-in-class inhibitor of FB, has proceeded to phase III studies, both in C5 inhibitor–treated and naïve patients. The results of the APPLY-PNH trial (NCT04558918), phase III RCT assessing iptacopan vs. eculizumab/ravulizumab in patients with PNH and anemia despite anti-C5 treatment, recently demonstrated efficacy and safety of iptacopan, achieving hemoglobin increase, transfusion avoidance, and resolution of extravascular hemolysis with adequate control of intravascular hemolysis. The APPOINT-PNH (NCT04820530) is a single-arm, phase III clinical trial with iptacopan in treatment-naïve patients. The first results of this study support the efficacy and safety of iptacopan [84,85]. These promising findings suggest that iptacopan monotherapy can become a potentially practice-changing outpatient and preferred therapeutic option for patients with hemolytic PNH.

**Table 2:** Complement inhibitors approved or in phase II/III trials for the treatment of PNH.

Terminal Complement Inhibitors								
Drug	Target	Class	Posology	Clinical Trials			Approval	Refs.
				Phase	Trial	Endpoints		
Eculizumab	C5	mAb	IV/2 wks	Phase III, completed	NCT00122330 (TRIUMPH)	Hb, PRBC transf.	FDA & EMA (2007)	[63,64]
					NCT00130000 (SHEPERD)	LDH		
Ravulizumab	C5	mAb	IV/8 wks SC form/ wk	Phase III, completed	NCT03056040	LDH	FDA (2018) EMA (2019)	[68,69]
					NCT02946463	LDH, TA		
Crovalimab	C5	mAb	SC/4 wks	Phase III, ongoing	NCT04432584 (COMMODORE 1)	Hb, reticulocyte count, haptoglobin	-	[72,73,74]
					NCT04434092 (COMMODORE 2)	LDH, TA		
					NCT04654468 (COMMODORE 3)	LDH, TA		
Pozelimab	C5	mAb	Combination: pozelimab (SC/wk) with cemdiseran (SC/variable)	Phase III, ongoing	NCT05133531 (ACCESS-1)	LDH, TA	-	-
Cemdiseran	C5	siRNA			NCT05131204 (ACCESS-2)	LDH		
**Proximal Complement Inhibitors**								
**Drug**	**Target**	**Class**	**Posology**	**Clinical Trials**			**Approval**	**Refs.**
				**Phase**	**Trials**	**Endpoints**		
Pegcetacoplan	C3/C3b	Peptide inhibitor, pegylated	SC twice/wk	Phase III, completed	NCT03500549 (PEGASUS)	Hb	FDA & EMA (2021)	[77,78]
					NCT04085601 (PRINCE)	Hb, LDH		
KP104	C5/C3b	Bi-functional mAb	SC/variable	Phase II, ongoing	NCT05476887	Hb	-	-
Danicopan	FD	Small molecule inhibitor	Oral TID, combined with eculizumab or ravulixumab	Phase III, ongoing	NCT04469465 (ALPHA)	Hb	-	[80]
Vemircopan	FD	Small molecule inhibitor	Oral BID	Phase II, ongoing	NCT04170023	Hb	-	[82]
BCX9930	FD	Small molecule inhibitor	Oral BID	Phase II, discontinued	NCT05116774 (REDEEM-1)	Hb	-	[83]
					NCT05116787 (REDEEM2)	Hb		
Iptacopan	FB	Small molecule inhibitor	Oral BID	Phase III, completed	NCT04558918 (APPLY-PNH)	Hb	-	[84,85]
					NCT04820530 (APPOINT-PNH)	Hb		

Abbreviations: BID: two times a day; EMA: European Medicines Agency; FDA: Food and Drug Administration; Hb: hemoglobin stabilization or increase in the absence of transfusions; IV: intravenous; LDH: lactate deshydrogenase levels; mAb: monoclonal antibody; PRBC: packed red blood cells; SC: subcutaneous; siRNA: small-interfering RNA; TA: transfusion avoidance; TID: three times a day; wk: week.

## 4. Conclusions

Many questions about the onset and course of PNH remain unanswered, such as the expansion after successful immunosuppressive therapy for AA, the tight relationship with AA and not with other autoimmune diseases, and, on the other hand, the cases arising without a previous phase of AA.

Although we are still halfway to understanding the pathophysiology of PNH, huge progress in the characterization of the disease and its immunologic environment has allowed for the formulation of several new theories and the refining and enriching of the canonical ones.

One can also embrace the possibility of the cooperation of multiple mechanisms and different ways of selection of the PNH clone, which can account for the biodiversity of disease phenotype and uncommon phenomena, such as PNH spontaneous remissions. The reliability of this last scenario seems to be supported by the high frequency, during the disease course, of additional mutations in *PIGA* cells and the subsequent arising of new clones (*PIGA* mosaicism, detected in at least 20% of patients), which reflect the variability and dynamicity of PNH.

Finally, for cases with a PNH and aplastic anemia overlap, the treatment follows that of bone marrow failure, while for those with the classic, hemolytic form of PNH, complement inhibitors are indicated. Eculizumab revolutionized the natural history of the disease, but several points of improvement are still unmet. Novel inhibitors targeting C5 and particularly proximal complement factors have recently emerged, and they are (or will soon be) enlarging the PNH therapeutic armamentarium to improve hemoglobin responses, the control of both residual and breakthrough hemolysis, and/or patients’ quality of life.

## Figures and Tables

**Table 1:** Overview of the main findings of the papers focusing on HLA expression in PNH patients.

Findings	Population	Ref.
High expression of class I fragment A*2501-Cw*1203-B*1801 and class II fragments DRB1*1501-DQB1*0602 and DRB1*0401-DQB1*0301 in PNH patients. In particular, association of A*2501-Cw*1203-B*1801 haplotype with AA/PNH and DRB1*1501-DQB1*0602 haplotype with dn/PNH.	44 PNH patients (9 AA/PNH, 31 de novo PNH, 4 unclassified PNH) and 200 ethnically matched controls.	[49]
Increased frequency of HLA class I alleles A*0201, B*1402, and Cw*0802, and HLA class II DRB1*1501 with the linked DQB1*0602 and DRB1*01 with the linked DQB1*0501 alleles in PNH patients. Significant increase in B*1402, Cw*0802 and A*33, B*1402, Cw*0802, DRB1*0102, DQB1* haplotype in PNH patients.	42 PNH patients and 301 controls of the same ethnic origin.	[51]
Increased expression of HLA-A*0206 in PNH patients.	78 patients (24 PNH, 32 AA, 22 MDS) and 371 controls of the same ethnic origin.	[53]
High incidence of HLA-DR2 alleles in patients with a PNH clone, in particular, in frankly hemolytic PNH and in PNH associated with bone marrow failure.	260 AA patients (48 with PNH clone), 139 MDS patients (29 with PNH clone), 93 patients with expanded PNH clone (48 AA/PNH, 29 MDS/PNH), and 46 patients with hemolytic PNH.	[54]
Increased frequency of DRB1*1501, DQA1*0102, and DQB1*0602 alleles and HLA-DRB1*1501-DQA1*0102-DQB1*0602 haplotype in PNH patients.	21 PNH patients, 21 AA patients, and 916 ethnically matched controls.	[52]
High frequency of DRB1*15:01 and DQB1*06:02 alleles in PNH, especially in PNH without AA. Association of B*18:01 allele with AA/PNH. Suggested protective role for A*24:02 allele.	13 AA/PNH patients, 33 de novo/PNH patients, 4 unclassified PNH patients, and 200 ethnically matched controls.	[50]

Abbreviations: AA, aplastic anemia; PNH, paroxysmal nocturnal hemoglobinuria; MDS, myelodysplastic syndromes; HLA, human leukocyte antigens.

## Data Availability

Not applicable.
